# A computer-assisted recording, diagnosis and management of the medically ill system for use in the intensive care unit: A preliminary report

**DOI:** 10.4103/0972-5229.58538

**Published:** 2009

**Authors:** George John, John Victor Peter, Binila Chacko, Kishore Pichamuthu, Aparajita Rao, K. Subbalakshmi, Kavitha Elizabeth George, Sawan Kumar Agarwal, S. Margret Anouncia, Ebenezer Sunderraj, Arul Siromoney

**Affiliations:** **From:** Medical Intensive Care Unit, Christian Medical College Hospital, Medical Intensive Care group, Vellore, India; 1 Vellore Institute of Technology (VIT) Software team, Vellore, India; 2 Information Technology Advisory Committee, Christian Medical College, Vellore, India; 3 Professor, Anna University, Chennai, India

**Keywords:** Alerts, alpha testing, checklist, decision support system, computer-assisted recording, diagnosis and management of the medically-ill

## Abstract

**Background:**

Computerized medical information systems have been popularized over the last two decades to improve quality and safety, and for decreasing medical errors.

**Aim:**

To develop a clinician-friendly computer-based support system in the intensive care unit (ICU) that incorporates recording, reminders, alerts, checklists and diagnostic differentials for common conditions encountered in critical care.

**Materials and Methods:**

This project was carried out at the Medical ICU CMC Hospital, Vellore, in collaboration with the Computer Science Department, VIT University. The first phase was to design and develop monitoring and medication sheets. Terminologies such as checklists (intervention list that pops up at defined times for all patients), reminders (intervention unique to each patient) and alerts (time-based, value-based, trend-based) were defined. The diagnostic and intervention bundles were characterized in the second phase. The accuracy and reliability of the software to generate alerts, reminders and diagnoses was tested in the third phase. The fourth phase will be to integrate this with the hospital information system and the bedside monitors.

**Results:**

Alpha testing was performed using six scenarios written by intensivists. The software generated real-time alerts and reminders and provided diagnostic differentials relevant to critical care. Predefined interventions for each diagnostic possibility appeared as pop-ups. Problems identified during alpha testing were rectified prior to beta testing.

**Conclusions:**

The use of a computer-assisted monitoring, recording and diagnostic system appears promising. It is envisaged that further software refinements following beta testing would facilitate the improvement of quality and safety in the critical care environment.

## Introduction

Critical Care Services in modern medicine play a vital role in delivering prompt, appropriate and adequate care to acutely ill patients. Acutely ill patients present with diverse pathophysiological derangements that require constant monitoring with rapid and repetitive interventions. These interventions often involve complex multi-modal approaches, all of which need to be seamlessly integrated in order to optimize outcome. Whilst some of these interventions/activities would be planned activities, others would include both initiated and reactive activities.[1] Planned activities could be scheduled routine (checklist) activities (bed position, deep venous thrombosis (DVT) and gastrointestinal (GI) prophylaxis) or standing orders unique to each patient (administration of a drug, replacement of fluid). Initiated activities are those that are not an integral part of routine treatment (e.g. placement of an arterial line) whilst reactive activities include activities in direct response to changes in the patients' clinical status.[1] It has been observed that, on an average, 178 such patient activities occur in a 24-h period for every patient in an intensive care unit (ICU). Of these, 84% of activities are performed by a single nurse, whilst only a small proportion is directly performed by physicians.[1] The sheer magnitude of tasks that need to be performed in a complex environment such as the ICU along with the amount of data that needs to be integrated and interpreted place the environment at considerable risk for errors.

Computerized systems have been gradually integrated into medical practice over the last few decades.[2] Systems termed as “Clinical Information Systems” (CISs) have been developed[3–6] in order to improve the quality of delivered services. These systems vary from those which provide basic functions to more complex and sophisticated “decision support systems”.[7] In a systematic review of 68 controlled trials of decision support systems published between 1974 and 1992, computer-based clinical decision support systems improved physician performance in 66%, particularly for drug dosing and preventive care but not for diagnosis.[8] In a more recent systematic review of 70 studies, decision support systems again significantly improved clinical practice in 68% of trials.[9] Some of the randomized trials included in this review[9] included computer-generated reminders for specific examination (e.g. breast examination) or tests (e.g. fecal occult blood test, cervical smear, mammography). A larger systematic review[10] incorporating 100 studies again found that such CISs improved practitioner performance in 64% of the studies with higher (*P* = 0.02) success rates with systems that automatically prompted users compared with those that required users to activate the system (73% vs. 47%).[10] These systematic reviews included studies predominantly in primary healthcare.

The use of such systems in the critical care environment is probably more relevant. In a simple approach, errors, particularly omissions, can be minimized in the complex ICU environment by the use of checklists,[11,12] that could remind the intensivist of routine tasks (e.g. DVT prophylaxis, GI prophylaxis, etc). These could be done manually, as a paper check or by electronic alerts at specified times (at admission or at a specified time each day). Capture of monitored data (pulse, blood pressure, respiration) directly on to electronic documentation sheets could potentially decrease the time spent by ICU nurses on paper documentation and channel the time to direct patient care.[13] Further, ICUs maintain a huge amount of data in several domains (cardiovascular, respiratory, neurological, etc) and employing a hybrid approach of case-based reasoning and rule-based reasoning could enhance patient care.[14] Benefits also include significant reductions in the rates of medication, intravenous therapy and ventilator incidents.[7]

However, the development of such integrated systems is challenging given the need for interaction between technologies and organizations.[15] Several systems which are developed have not been used clinically as they are not user-friendly and are primarily developed from an engineering perspective.[16] Thus, clinician involvement is crucial in the development of CISs to ensure a user-friendly system that is applicable to several clinical settings and situations. In this study, we present an integrated system that was designed to incorporate functions of recording, reminding, alerting, checklists and diagnostic alerts for common conditions encountered in critical care practice. This joint project, developed by intensivists and computer software students and teachers as a project, has been given the acronym - CARDAMOM (Computer-Assisted Recording, Diagnosis and Management of the Medically-ill).

## Materials and Methods

This project was carried out at the Christian Medical College Hospital, Vellore, a tertiary care teaching hospital in South India, in collaboration with the Computer Science Department, Vellore Institute of Technology over a six-month period (June-November 2008). The project was designed and developed in four phases. In each of the phases, a detailed discussion occurred between the intensivists and the computer software developers on the requirements in the critical care environment. We did not use any predefined or previously published system approach as we were aware of the inherent problems and difficulties in adapting such systems to local needs and practice. The approach was purely driven by clinical requirements and these requirements were expressed to the information technology (IT) students and professionals at each stage of the development. In the first week of the project, the computer software students spent several hours in the critical care unit trying to understand how data was collected and recorded in the monitoring sheets that are presently used in the ICU.

Following this, in the first phase, monitoring and medication sheets were designed and developed and several terminologies were defined. The second phase was devoted to the development of diagnostics as well as the intervention bundle. The third phase (still being tested at the time of submission of article) was real-time testing. The final phase of the study will involve interfacing the investigations and radiology images (PACS) available on our Hospital Information System (HIS) with the CARDAMOM system as well as capturing real-time monitoring data from the bedside monitors automatically.

### Phase 1 - Monitoring and medication sheets; definitions

The system was programmed such that data could be entered only after the hospital number specific to a patient and the length of the patient were entered. The length of the patient was mandatory as several calculations such as the tidal volume are based on the length.

**Figure 1:**
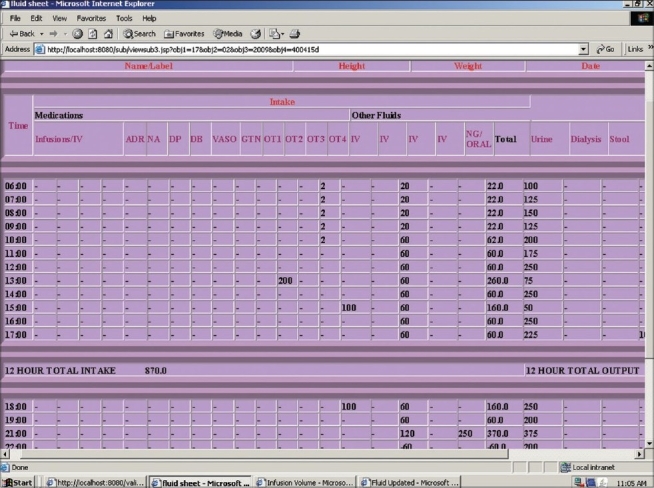
Picture showing hourly fluid balance chart

**Figure 2:**
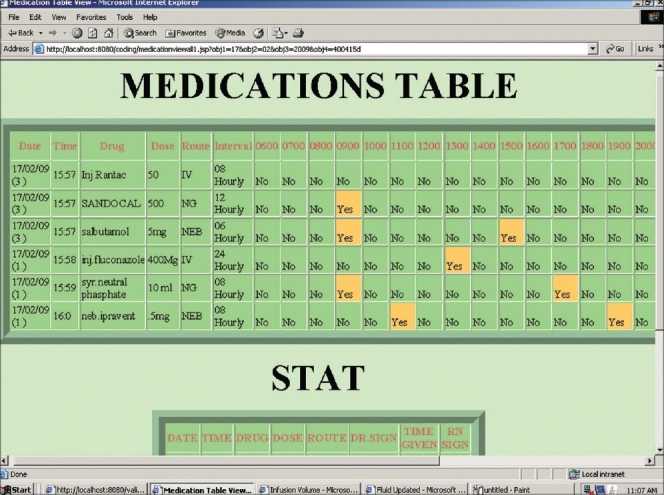
List of medications and the timing when it is to be administered

The user interface was designed to have the following modules:

- *Vital signs chart* that contained temperature, heart rate, respiratory rate, oxygen saturation (SpO_2_) and blood pressure. The blood pressures were recorded as systolic and diastolic pressures and the system calculated the mean arterial pressure. These parameters were entered on a time (at least hourly) basis. Results for these were displayed graphically. Ventilatory parameters such as FiO_2_, tidal volume, peak airway pressures and positive end-expiratory pressure (PEEP) settings were included for ventilated patients.
- *Fluid balance chart* that included hourly intake and output. The system calculated the total at 12 and 24 h along with cumulative fluid balance over the entire period of admission [Figure 1].
- *Investigation sheet* wherein all investigations (biochemical, hematological, microbiological and serological) pertaining to the patient could be entered manually. Refinement of this, to be done subsequently, would be to capture the data automatically from the Hospital Information System (see Phase 4).
- *Medication chart* containing medications at predefined intervals, infusions, fluids as well as STAT and PRN medications [Figure 2]. When the medications were entered along with the frequency, the time of administration of the drug was automatically generated based on predefined rules of drug administration (e.g. once daily drug at 8 am in the morning, subsequent medications on a regular basis over the 24-h period).

**Figure 3:**
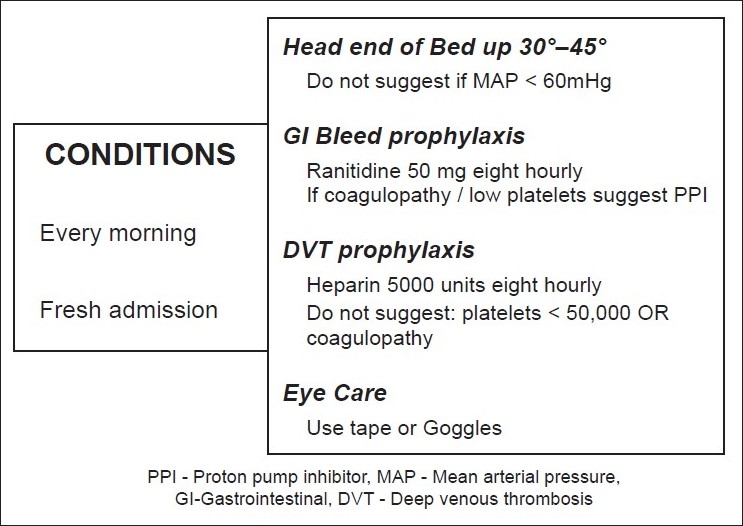
Checklist. For example, a checklist will pop up on the screen every day at 8 am and will remain on screen till each of the events are ticked off. For new patients, the same checklist will appear and each aspect needs to be acknowledged

Several terminologies were also defined during the first phase. *Checklists* were defined to be a list of interventions that pop up at defined times for all patients. This was not patient-specific. For example, for all ICU patients, a checklist would remind of GI bleed prophylaxis, DVT prophylaxis, propping up the patient at 30° head elevation etc [Figure 3]. On the other hand, *reminders* were time-based, unique to each patient and were entered by the treating intensivist. For example, a reminder would pop up about 15 min prior to a scheduled serum potassium level check. *Alerts* were categorized as *time*-*based* (as in the reminder above) or value-*based*, wherein an alert comes up when a predefined maximum or minimum value is crossed (e.g. when the SpO_2_ decreases to 80% a LO alert would come up). *Trend*-*based alerts* were defined for wide changes within the total bandwidth of the alert. For example, an increase or decrease from a previous value by 25% within the total bandwidth would trigger a trend-based alarm. An *alarm* specifies how the alert is displayed (either visually or by sound or a combination of both). A *diagnosis* was defined as a condition in which two or more criteria were fulfilled. An *intervention* was defined as a bundle of actions that were specific to a diagnosis.

Several problems encountered during this phase merit mention here. The first was the need for several meetings with the IT students and teachers to help understand medical terminologies as well as diagnostic possibilities. The second major problem was to define thresholds for alarms, based not only on absolute values but also on trends. It is well recognized that significant changes in vital parameters, occurring over a short period of time, even within the “normal range” may be of clinical importance. For example, a sudden increase in the heart rate from a baseline 60 beats/min to 100 beats/min may signify the onset of an infection, volume loss or other major catastrophe. However, when only a value-based alert is placed (alert outside of the normal range of 60 to 100), then such change within the normal range would not result in an alert. Thus, a trend-based alert was described. Third, the initial confusion about the difference between a reminder and a checklist was sorted out by precise definitions as stated in the text above.

### Information technology platform

Decisions regarding the types of IT platforms were taken. Windows XP and ORACLE 10 g were chosen to be the operating system and the database system respectively, since these are the existing platforms used in the hospital. After discussion, Java, JSP and HTML were the languages used and Apache Tomcat 5.5 was the web server.

### Phase 2 - Diagnostics and intervention bundle

**Figure 4:**
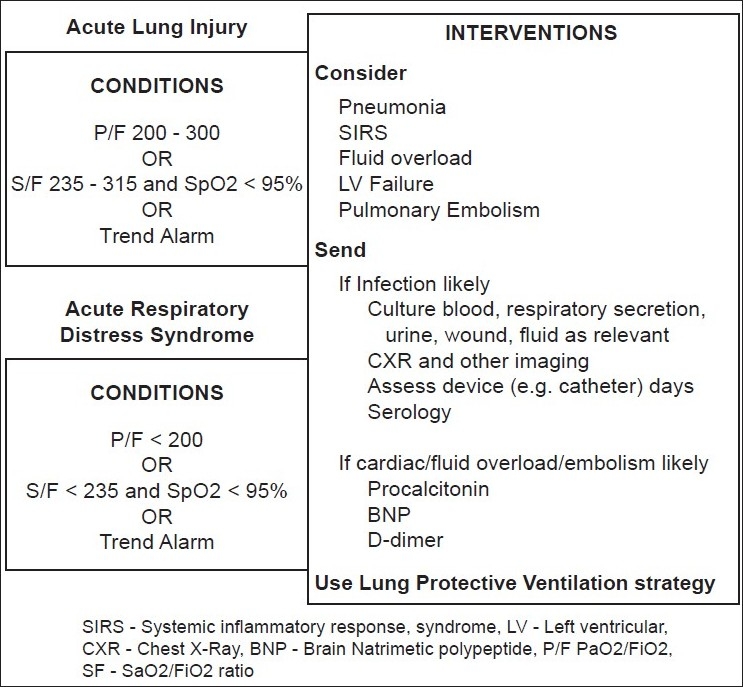
Example of a diagnostic and intervention bundle. A diagnostic bundle (left boxes) would be activated if certain conditions are fulfilled. For the intervention bundle, a set of interventions would pop up. These interventions were decided by a panel of intensivists after several discussions

The diagnostics was developed to provide the intensivist a list of possible diagnoses when two or more parameters were beyond acceptable limits. The diagnostic list contained only those entities that could be reasonably diagnosed based on parameters in the modules. For example, the diagnostic list does not include encephalitis, but includes shock and pulmonary embolism. The list of diagnoses that were included is summarized in Table 1. The intervention bundle consisted of a series of actions specific to the diagnosis [Figure 4]. The display of this bundle is triggered by the diagnosis. For example, in a patient with a diagnostic alert of “systemic inflammatory response syndrome (SIRS),” the intervention bundle would suggest to take appropriate cultures, check central venous pressure and consider fluid resuscitation or start vasoactive agents. These specific interventions were listed following a consensus by four intensivists and based on the best available evidence with available resources.

**Table 1:** List of common intensive care unit diagnoses included in the diagnostic bundle

Acute fluid loss	Systemic inflammatory response syndrome
Acute blood loss	Sepsis
Acute lung injury	Severe sepsis/Septic shock
Acute respiratory distress syndrome	Shock, including cardiogenic shock
Obstructive airways disease	Fluid overload
Atelectasis/Collapse	Left heart failure
Pulmonary embolism	Disseminated intravascular coagulation
Pneumothorax	Thrombotic thrombocytopenic purpura
Deteriorating Glasgow coma score	Hemolytic uremic syndrome
Acute Kidney Injury RIFLE Class	Heparin-induced thrombocytopenia

There was an in-depth discussion regarding the number of criteria that needed to be met in order to trigger a diagnostic possibility. Although we initially felt that even a single criterion should trigger a diagnostic possibility, we subsequently realized that such a threshold would continuously raise numerous diagnostic possibilities in the critical care environment where physiological derangements are the norm. We thus modified the diagnostic list to be triggered when two or more criteria were met. Each diagnostic list, triggered by a set of parameters, was carefully scrutinized to ensure that it was inclusive rather than exclusive and also listed in order of relevance (see results below). The intervention bundles specific to each of these diagnoses were worked out independently by each intensivist and subsequently pooled to ensure that all aspects of intervention were included.

### Phase 3 - Testing of software

The third phase of the project was testing the software. Testing of the software was conducted in two phases-the alpha and beta testing.[17,18] The alpha test is the process of testing the product amongst the team to confirm that the product works. This was performed by the use of six scenarios written by intensivists on the regular ICU monitoring sheets. The problems pertaining to documentation, checklists, diagnostics or intervention were evaluated. Some modifications were made to ensure that all diagnostic possibilities that were trigged by a set of parameters were listed. Other problems encountered during this phase are discussed in the results section below.

The beta test consisted of real-time testing in the ICU on patients actually admitted to the unit. Only the alpha testing was part of this initial study. The beta testing and interfacing of investigations were in progress at the time of submission.

### Phase 4 - Interfacing of investigations and monitoring data

Interfacing of investigations that are available online on the HIS with the CARDAMOM software would be the final phase of the project (Proposed). The investigations would include hematology, pathology and biochemical tests, microbiology, images as well as arterial blood gas estimations. In addition, during this phase, real-time monitoring data from the bedside monitors would also be captured directly and integrated with the CARDAMOM software.

## Results

Alpha testing was done in two stages. In the first stage, two case scenarios were constructed by an intensivist and this was analyzed by the CARDAMOM software. Feedback regarding the performance in terms of accuracy and reliability was given and modifications were made to the software before the second stage of alpha testing with four more case scenarios.

It was observed that the alerts and reminders occurred appropriately in real-time. However, it was noted, after the first two scenarios, that there was a problem in the diagnostic list in that the differential diagnoses were not presented in the appropriate order (with the most likely diagnosis being the first). It appeared that for every diagnostic category, the main positive features were matched against the clinical presentation. For example, if diagnosis A needed four positive criteria (M, O, P, Q) for a perfect match but only three of the four criteria were met, a 75% probability was assigned to this diagnosis. However, if diagnosis B consisted of only two positive criteria (M, O) but other negative criteria (R, S, T) were not incorporated into the rule-based diagnosis, a 100% positive match (M and O present) put diagnosis B above diagnosis A.

**Figure 5:**
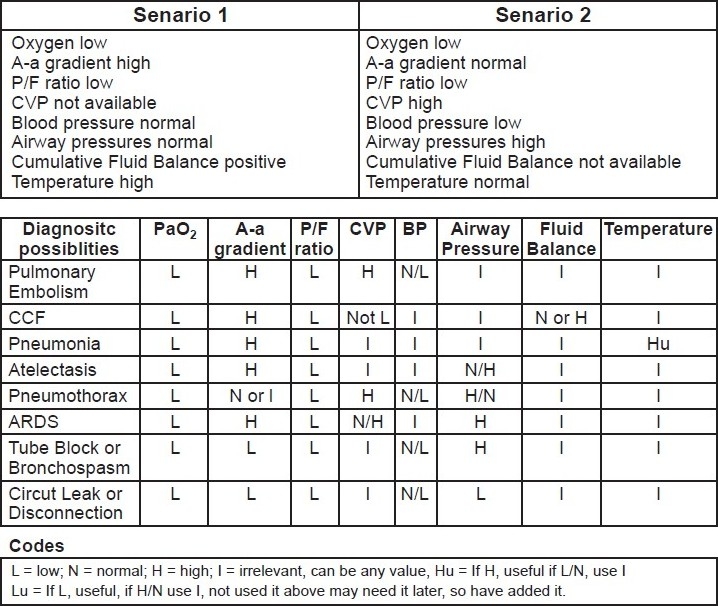
Interaction of positive and negative criteria in clinical scenarios. The most likely diagnosis for Scenario 1 is pneumonia and for Scenario 2 it is pneumothorax. There will be (and should be) other possibilities which are less matched but still to be considered in a lower order

In order to circumvent this problem, both positive as well as negative criteria were incorporated into the algorithm such that the list of differential diagnoses was narrowed down and the list generated was in a more clinically appropriate order. An example of such an interaction is summarized in the example in Figure 5.

After completion of all the six scenarios, a written feedback was provided to the software developers and the program was fine-tuned. This is now followed by beta-testing of the software in real-time in the ICU for a period of six months. This is currently under way.

## Discussion

Expert systems are being increasingly recognized as an important tool for clinicians. Expert systems or knowledge-based systems use computer programs that contain some of the subject-specific knowledge of one or more experts.[14] These CISs support a wide range of tasks ranging from simple reminders regarding routine tests to the more complex decision support systems that have the potential to enhance the quality and efficiency of treatment.[7,14] Such systems may play a vital role in a critical care environment since a large volume of data related to a single patient needs to be integrated over time. The complexity of critical care and the enormity of tasks that need to be performed for each patient[1] place the environment at risk for errors and preventable mishaps. Decision support systems that incorporate both routine functions such as checklists, alerts and reminders as well as diagnostic possibilities and suitable interventions thus score over the more basic CISs that are widely used currently. It is envisaged that such systems would increase efficiency and improve quality of care and outcomes.

The development of user-friendly tools has been limited by the lack of ‘clinician’ involvement. This has been recognized by the American Informatics Association (AMIA) and also reported in the Royal Australasian College of Physicians' newsletter[16] as a cause for failed systems. Another possible reason cited for failure is the predominantly ‘administrative’ (business) model with minimal clinical functionality.[16]

Our software program CARDAMOM has been developed by a team. Several meetings at regular intervals between the two groups of professionals occurred. Such discussions focused on the utility as well as applicability of the software not only in a tertiary level critical care environment but also at a secondary level hospital. At each stage of the development of the software the interface between computer experts and medical personnel ensured that the program was not only user-friendly but also clinically functional, catering to the needs of the Indian critical care patient.

Some limitations merit mention here. Alpha testing was done on six case scenarios that were constructed by the intensivists. This has not been previously validated. We felt that further validation of the diagnostic bundle should be done at the beta phase of the testing when real patients' data are entered, rather than using more case scenarios. Although some refinements have been made to the software following these scenarios, it is likely that more refinements may be required to make the diagnostic bundles more inclusive. Secondly, at present the software has not been enabled to capture data directly from the bedside monitors. These are currently entered manually at present. The investigations also need to be entered manually. However, in Phase 4 of this project, it is hoped that both these could be seamlessly integrated by capturing data directly from the bedside monitors as well as from the Hospital Information System.

The successful alpha testing of the software has resulted in the beta testing being initiated. The accuracy of the software to prompt real-time checklists, alerts and reminders is encouraging and would aid in initiating appropriate and timely treatment. It is hoped that the differential diagnoses lists that come up in relation to derangements in physiological parameters would alert the physician in training to the various clinical possibilities and serve as a learning tool and reduce errors. However, it must be remembered that when a diagnostic likelihood is based on the combination of positive and negative features, it may be impossible to derive an algorithm that will always accurately list diagnoses in the correct order. Thus, the software should necessarily provide an inclusive rather than an exclusive or prioritized list of differentials, that are relevant and of significance in a critical care setting, that would alert the physician to all diagnostic possibilities, particularly in times of intense stress when a diagnosis or a test might not be considered and missed out. It is with these objectives that this CARDAMOM software has been designed to “assist” the clinician and not “supplant” rational decision-making and prioritization by the physician.
